# Persistence and transgenerational effect of plant-mediated RNAi in aphids

**DOI:** 10.1093/jxb/eru450

**Published:** 2014-11-16

**Authors:** A. D. Coleman, R. H. M. Wouters, S. T. Mugford, S. A. Hogenhout

**Affiliations:** Department of Cell and Developmental Biology, John Innes Centre, Norwich Research Park, Norwich NR4 7UH, UK

**Keywords:** Aphid, dsRNA acquisition by feeding, effector, green peach aphid, Hemiptera, *Myzus persicae*, saliva, silencing, systemic movement, virulence and fecundity.

## Abstract

Plant-mediated RNA interference (RNAi) has been successfully used as a tool to study gene function in aphids. The persistence and transgenerational effects of plant-mediated RNAi in the green peach aphid (GPA) *Myzus persicae* were investigated, with a focus on three genes with different functions in the aphid. *Rack1* is a key component of various cellular processes inside aphids, while candidate effector genes *MpC002* and *MpPIntO2* (*Mp2*) modulate aphid–plant interactions. The gene sequences and functions did not affect RNAi-mediated down-regulation and persistence levels in the aphids. Maximal reduction of gene expression was ~70% and this was achieved at between 4 d and 8 d of exposure of the aphids to double-stranded RNA (dsRNA)-producing transgenic *Arabidopsis thaliana*. Moreover, gene expression levels returned to wild-type levels within ~6 d after removal of the aphids from the transgenic plants, indicating that a continuous supply of dsRNA is required to maintain the RNAi effect. Target genes were also down-regulated in nymphs born from mothers exposed to dsRNA-producing transgenic plants, and the RNAi effect lasted twice as long (12–14 d) in these nymphs. Investigations of the impact of RNAi over three generations of aphids revealed that aphids reared on dsMpC002 transgenic plants experienced a 60% decline in aphid reproduction levels compared with a 40% decline of aphids reared on dsRack1 and dsMpPIntO2 plants. In a field setting, a reduction of the aphid reproduction by 40–60% would dramatically decrease aphid population growth, contributing to a substantial reduction in agricultural losses.

## Highlight

This work shows that the transgenerational effect of RNA interference in the green peach aphid *Myzus persicae* contributes to a 60% decline in aphid population growth.

## Introduction

RNA interference (RNAi) is a cellular process used by animals, plants, and fungi as a means of post-transcriptional gene regulation to maintain normal growth and development, and mediates defence responses against viruses or transposable elements (Hannon, 2002). Over the past 15 years, RNAi has been successfully exploited as a reverse-genetics tool to study gene function in various organisms. The green peach aphid (GPA) *Myzus persicae* is an important agricultural pest and is developing into a valuable model system to investigate aphid gene function using RNAi-based techniques. Vast amounts of genomics data are being generated for aphid systems, increasing the usefulness of RNAi approaches. The pea aphid (*Acyrthosiphon pisum*) genome has been published (Richards *et al.*, 2010), and GPA clone O and G006 are being sequenced as a collaboration between UK, French, and US research teams (http://www.aphidbase.com/myzusdb). Functional genomics tools, such as RNAi, are essential to make use of the increasing availability of aphid genome and transcriptome sequence data.

The RNAi process relies on double-stranded RNA (dsRNA) precursors, which can specifically lower the transcript abundance of a target gene when injected into an organism or when introduced into cultured cells (Fire *et al.*, 1998). RNAi involves the cleavage of the dsRNA precursors into small-interfering RNA (siRNA) of ~21–23 nucleotides by the enzyme Dicer-2 (Dcr-2) (Meister and Tuschl, 2004). These siRNAs are then incorporated into an RNA-induced silencing complex (RISC). Argonaute-2 (Ago-2), the catalytic component of RISC, uses the siRNA as a template to recognize and degrade the complementary mRNA (Meister and Tuschl, 2004). RNAi therefore suppresses gene expression via highly specific depletion of target transcripts.

RNAi-mediated gene down-regulation can be achieved in aphids through direct injection of dsRNA or siRNA into aphid haemolymph (Mutti *et al.*, 2006, 2008; Jaubert-Possamai *et al.*, 2007). Feeding of dsRNA from an artificial diet also suppresses expression of the corresponding aphid gene (Shakesby *et al.*, 2009; Whyard *et al.*, 2009). The feasibility of using plant-mediated RNAi to initiate down-regulation of gene targets in GPA has previously been shown (Pitino *et al.*, 2011; Pitino and Hogenhout, 2013). Other research groups have also used plant-mediated RNAi to reduce gene expression in GPA (Bhatia *et al.*, 2012; Guo *et al.*, 2014).

It was demonstrated that plant-mediated RNAi of three genes, *Rack1*, *MpC002*, and *MpPIntO2* (also known as *Mp2*), reduced fecundity of GPA (Pitino *et al.*, 2011; Pitino and Hogenhout, 2013). Plant-mediated RNAi of another target gene, *MpPIntO1* (also known as *Mp1*), also successful depleted the transcript abundance but did not result in reduced fecundity of the aphids (Pitino and Hogenhout, 2013). Rack1 is a key mediator of various cellular pathways: it is involved in shuttling and anchoring proteins in the cell and has been shown to interact with the ribosomal machinery and cell surface receptors (Adams *et al.*, 2011). It is therefore involved in diverse physiological processes such as development, cell migration, and circadian rhythm (Adams *et al.*, 2011). MpC002 is the *M. persicae* homologue of *Acyrthosiphon pisum* C002 (Mutti *et al.*, 2006, 2008), which has previously been shown to be important in aphid interaction with the host plant (Mutti *et al.*, 2008; Bos *et al.*, 2010). MpPInt02 (Mp2) is a protein that is produced in the GPA salivary glands (Bos *et al.*, 2010; Pitino and Hogenhout, 2013). Both MpC002 and MpPIntO2 are candidate effector proteins that affect plant colonization of GPA. Ectopic expression of genes encoding these effectors in the plant *Arabidopsis thaliana* promotes GPA reproduction on these plants, while down-regulation of these genes by plant-mediated RNAi in the aphids reduces GPA reproduction (Pitino *et al.*, 2011; Pitino and Hogenhout, 2013).

The mechanisms underlying plant-mediated RNAi in aphids and the potential of the technology towards the control of aphid pests are still in the early stages of development. In this study, the aim was to investigate the persistence of RNAi in GPA and the longer term effects of plant-mediated RNAi on aphid performance. The time taken to achieve optimal gene down-regulation after exposure to dsRNA, the duration of the gene down-regulation upon removal of aphids from the dsRNA source, and whether the RNAi effect is transferred to the aphid progeny were investigated. Interestingly, down-regulation of target genes was also observed in nymphs born from mothers exposed to plant dsRNAs, thus generating a germline effect. Moreover, a dramatic reduction in aphid population growth was observed upon continuous exposure to various dsRNA sources.

## Materials and methods

### Plant and insect growth/maintenance conditions

The GPA lineage used in this work is *Myzus persicae* RRes (genotype O) (Bos *et al.*, 2010). GPA were reared on Chinese cabbage (*Brassica rapa*), and plants and insects were maintained in custom-built acrylic cages located in controlled environment conditions with a 14h day (90 μmol m^–2^ s^–1^ at 18 °C) and a 10h night (15 °C) photoperiod.

### Plants used in this study

The generation of all transgenic plants used in this study has been previously described (Pitino *et al.*, 2011; Pitino and Hogenhout, 2013). Briefly, fragments corresponding to *GFP* (green fluorescent protein), or GPA *Rack1*, *MpC002*, or *MpPIntO2* (*Mp2*) transcripts were amplified and cloned into the pJawohl8-RNAi binary vector for expression of dsRNAs under control of a 35S promoter. Experiments were conducted with homozygous T_3_ lines that carry single transgenes (Pitino *et al.*, 2011; Pitino and Hogenhout, 2013).

### Preparation of RNAi insects

All transgenic and wild-type *A. thaliana* used in insectary bioassays were prepared similarly to allow generation of evenly aged test insects. *Arabidopsis thaliana* were grown in medium-grade compost (Scotts Levington F2) and initially maintained under controlled environment conditions of 18 °C, 10h day, 60% humidity. At 10–14 d after sowing, plants were transferred individually to single wells (5cm^3^) of 24-well trays. Four-week-old plants were individually transferred to 1 litre round pots (13cm diameter, 10cm tall) and covered by pushing round, transparent-plastic experimental cages (10cm diameter, 15cm tall; Jetran tubing, Bell Packaging Ltd, UK) with a gauze-covered plastic lid into the soil of plant pots to contain the aphids. Experiments were replicated three times to generate three biological replicates. Each biological replicate consisted of three technical replicates of at least three plants of each dsGFP, dsRack1, dsMpC002, and dsMpPIntO2 transgenic line exposed to GPA adults from the stock colony for 2 d, after which all adults were removed, leaving five 0- to 2-day-old nymphs per plant to be used as the experimental insects. All *A. thaliana* whole-plant bioassays with GPA were performed under controlled environment conditions of 8h day (90 μmol m^–2^ s^–1^ at 18 °C) and 16h night (16 °C).

### qRT-PCR analyses to investigate down-regulation of aphid target genes

Total RNA was extracted from GPA exposed to test plants using TRIzol reagent (Life Technologies, Paisley, UK). DNA was removed by treating RNA extractions with RNase-free DNase (QIAGEN, West Sussex, UK) then purified with QIAamp columns (QIAGEN). GPA mRNA was also obtained using a Dynabeads mRNA DIRECT kit (Life Technologies) according to the manufacturer’s instructions. First-strand cDNA was synthesized at 37 °C from RNA isolations using M-MLV (Invitrogen) reverse transcriptase according to the manufacturer’s instructions. Quantitaive real-time PCRs (qRT-PCRs) were laid out in 96-well plates (Thermo Scientific), with each sample represented by the gene of interest and two reference genes [L27 and glyceraldehyde phosphate dehydrogenase (GAPDH)] as determined by GeNORM (see below). Two or three technical replicates were included for each cDNA–primer combination. Individual reactions contained 3 μl of cDNA, 0.5 μl of specific primers (forward and reverse primer at 10 pmol ml^–1^), and 10 μl of 2× SYBR Green (Sigma-Aldrich) in a final volume of 20 μl. Plates were sealed using adhesive PCR Film (Thermo Scientific). Plates were run in a CFX connect™ machine (Bio-Rad) at 90 °C for 3min, followed by 40 cycles of 95 °C for 30 s, 60 °C for 30 s, 72 °C for 30 s, and finally 10min at 72 °C. The SYBR-specific fluorophore was quantified during the reaction by the instrument.

### Selection of qRT-PCR reference genes

To determine which reference genes were most stable under the experimental conditions and also the optimum number to use, a GeNORM analysis was performed using Biogazelle qBasePLUS software (Biogazelle, Zwijnaarde, Belgium). The expression of eight potential reference genes was measured in GPA at different ages exposed to each of the dsRNA-expressing plants. From this, the two reference genes shown to be sufficient for the present experiments and the most stable in GPA of different ages and after different dsRNA treatments were *L27* and *GAPDH*.

### Long-term population assay

A GPA population was established on 4-week-old dsRack1, dsMpPIntO2, dsMpC002, or dsGFP plants, and the insects were counted over successive weeks. Four plants per line were replanted with equidistant spacing in custom-made experimental cages. Cages consisted of a black plastic tray (30cm width, 45cm length, 5cm depth) filled with medium-grade compost (Scotts Levington F2), and a clear-plastic lid (30cm width, 45cm length, 20cm height) with mesh on top to allow ventilation; all sides were sealed with adhesive tape. A single, 0- to 2-day-old GPA nymph was left on each plant to establish a population. Adults and nymphs were counted at 2, 3, and 4 weeks post-introduction of aphids. This experiment was repeated to give three biological replicates.

### Statistical analyses

All statistical analyses were performed using GenStat statistical software (15th edition, VSNi Ltd, Hemel Hempstead, UK). To perform statistical analyses on qRT-PCR data, threshold cycle [C(t)] values were calculated using CFX manager (Bio-Rad). Ct values for the two reference genes (*L27* and *GAPDH*) and target genes (*Rack1*, *MpC002*, and *MpPIntO2*) were calculated for each of the three technical replicates and averaged. Then the mean value generated from the two reference genes was used to calculate the relative gene expression of the target genes using the 2^–ΔΔCT^ method as previously described by Livak and Schmittgen (2001). The values for three biological replicates (*n*=3) were normalized to the expression level of dsGFP control aphids set at 1 and analysed using Student’s *t-*test to determine whether the mean normalized transcript levels of target genes for GPA fed on transgenic plants expressing dsRNA corresponding to the target gene were significantly different from those of aphids fed on dsGFP (control) plants. Individual *t-*tests were performed between dsGFP and each other dsRNA treatment for each time period separately. A generalized linear model (GLM) was also used to determine differences between specific RNAi targets (i.e. *Rack1*, *MpC002*, or *MpPIntO2*) or between experimental replicates. Means for biological replicates and treatments at each time point were compared using *t-*probabilities calculated by the GLM (GenStat 15th edition, VSNi Ltd).

## Results

### Maximal level of gene down-regulation in GPA occurs within 8 d of exposure to RNAi plants

Previous experiments demonstrated down-regulation of aphid target genes after 16 d feeding on transgenic *A. thaliana* producing dsRNA corresponding to aphid genes (Pitino *et al.*, 2011). However, maximal down-regulation of aphid target genes may occur earlier than 16 d, and it is not known how long aphid genes remain suppressed after removal of the aphid from the dsRNA source. To investigate this, relative levels of the *Rack1*, *MpPIntO2*, and *MpC002* transcripts were monitored over time in aphids feeding on plants expressing the corresponding dsRNA constructs.

**Fig. 1.**
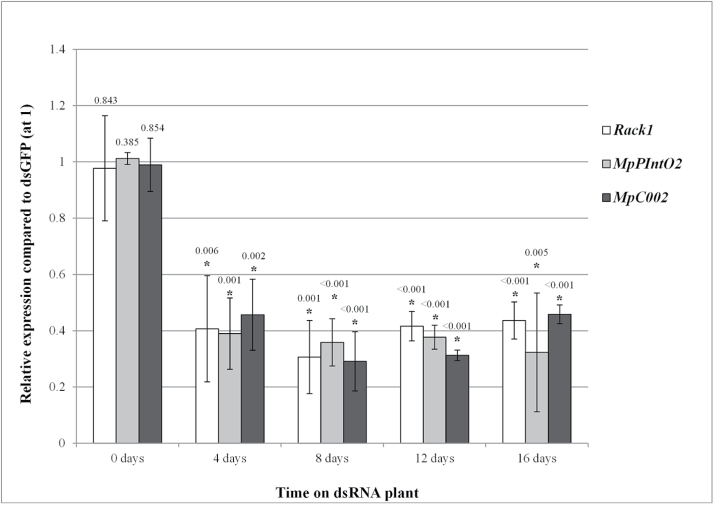
Maximal down-regulation of target genes targeted by plant-mediated RNAi occurs within 8 d post-first day exposure to transgenic *Arabidopsis thaliana* plants producing dsRNAs. GPA were reared on dsRNA-expressing plants over a 16 d time series. Aphids were harvested at 0, 4, 8, 12, and 16 d to test for target gene down-regulation by qRT-PCR. Bars represent average expression of the corresponding target gene at each time point for aphids reared on dsRack1 (white), dsMpPIntO2 (grey), or dsMpC002 (black) compared with aphids reared on dsGFP. Data represent mean expression levels ±SD for each target gene at each time point for three biological replicates. Relative target gene expression values were normalized to the expression level of dsGFP aphids set at 1. Asterisk indicates significant difference compared with the dsGFP control (Student’s *t-*test, *n*=3, *P*<0.05), and *P*-values (to three decimal places) corresponding to individual data points are displayed above each bar.

Single transgenic plants producing dsRack1, dsMpPIntO2, dsMpC002, or dsGFP plants were seeded with GPA nymphs of 0–2 d old. Then three batches (serving as individual technical replicates within an experiment) of five insects per dsRNA treatment were sampled from these plants immediately (day 0) and at 4 d intervals over 16 d and processed for qRT-PCR analyses to assess the mean level of *Rack1*, *MpPIntO2*, or *MpC002* down-regulation relative to dsGFP-fed aphids. As expected, the target genes were not down-regulated in aphids harvested at day 0 (Student’s *t-*test, *n*=3, *P*>0.05), whereas up to 60% down-regulation of the target genes was observed after 4 d and up to 70% down-regulation at 8 d (Student’s *t-*test, *n*=3, *P*<0.0056) (Fig. 1). The level of down-regulation remained constant at 50–70% to the end of the experiment at 16 d (Student’s *t-*test, *n*=3, *P*<0.0056) (Fig. 1). No significant differences in the levels of down-regulation between the target genes were observed (GLM, *n*=3, *P*>0.05), indicating that plant-mediated RNAi is sequence independent.

### RNAi effect disappears at 6 d upon removal of GPA from dsRNA plants

**Fig. 2.**
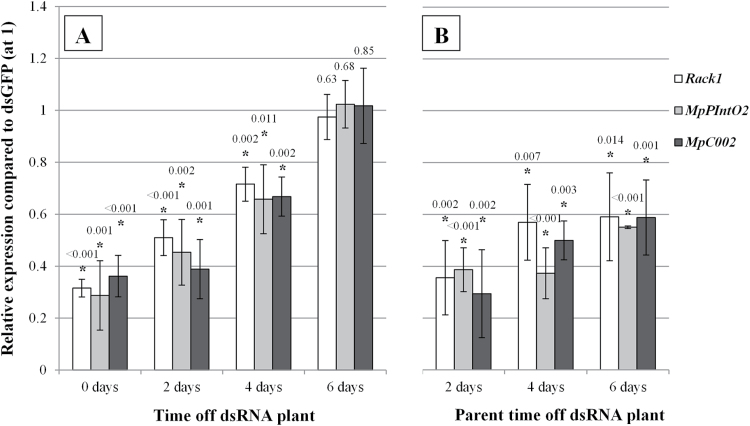
Continuous exposure to dsRNAs is required to attain maximal levels of down-regulation of aphid genes targeted by plant-mediated RNAi in adult aphids and progeny of these aphids. RNAi GPA were transferred to Col-0 plants then harvested at 0, 2, 4, and 6 d to test for target gene down-regulation by qRT-PCR. Second-generation nymphs were born from RNAi-exposed first-generation adults within 0–2 d of transfer to Col-0 plants. These nymphs were also collected at 2, 4, and 6 d. Relative expression of *Rack1*, *MpPIntO2*, or *MpC002* was determined in adults (A) fed on dsRNA(target) and their nymphs (B) compared with dsGFP-fed equivalents. Bars represent average expression of the corresponding target gene at each time point for aphids reared on dsRack1 (white), dsMpPIntO2 (grey), or dsMpC002 (black) compared with aphids reared on dsGFP. Data represent mean expression levels ±SD for each target gene at each time point for three biological replicates. Relative target gene expression values were normalized to the expression level of dsGFP aphids set at 1. An asterisk indicates a significant difference compared with the dsGFP control (Student’s *t-*test, *n*=3, *P*<0.05), and *P*-values (to three decimal places) corresponding to individual data points are displayed above each bar.

Next, it was assessed whether target gene down-regulation in GPA reverts to normal levels after removal of aphids from the dsRNA-expressing plants. Aphids of 0–2 d old were exposed to the RNAi plants for 8 d to attain maximal down-regulation at 50–70% of *Rack1, MpPIntO2*, and *MpC002* (Fig. 1) and then removed from the RNAi source by placement on wild-type Col-0 plants. Subsequently, three batches of five insects were sampled immediately (day 0) and at 2 d intervals. The qRT-PCR data revealed 50–70% down-regulation at day 0 (Fig. 2A), in agreement with previous data (Fig. 1). *Rack1*, *MpPIntO2*, and *MpC002* expression levels increased in GPA reared for 2 d and 4 d (Student’s *t-*test, *n*=3, *P*<0.011) on wild-type Col-0 plants, increasing further in a linear fashion over 6 d, at which time point no differences in target gene expression levels were observed compared with aphids exposed to the dsGFP treatment (Student’s *t-*test, *n*=3, *P*>0.63) (Fig. 2A). No significant difference was found between dsRack1, dsMpPIntO2, and dsMpC002 treatments at each time point (GLM, *n*=3, *P*>0.05), indicating that this recovery of gene expression is a general phenomenon.

### RNAi effect is transferred to GPA progeny in which down-regulation is sustained for longer

Embryos develop in aphid females exposed to plant-mediated RNAi. Therefore, it was investigated whether the RNAi effect is transferred to the aphid progeny. To this end, the progeny (second generation) derived from aphids exposed to the transgenic plants examined in Fig. 2A (first generation) were processed for qRT-PCR at 2 d intervals. Because the first-generation females were transferred to non-transgenic *Arabidopsis* plants, the second-generation females were born on the non-transgenic plants and hence were not exposed to the dsRNA source via the plant host. Up to 75% down-regulation of target genes was found in the second-generation aphids (Student’s *t-*test, *n*=3, *P*<0.0068) (Fig. 2B), indicating that the RNAi effect had transferred from mothers to the embryos. Interestingly, the target gene down-regulation lasted longer in the second-generation aphids than in their mothers, since at 6 d after removing the parent insect from dsRNA plants the second-generation aphids showed up to 40% down-regulation of target genes (Fig. 2B) (Student’s *t-*test, *n*=3, *P*<0.014), while adults had normal levels of target gene expression (Fig. 2A). Again, no significant differences were found between dsRack1, dsMpPIntO2, and dsMpC002 treatments at each time point (GLM, *n*=3, *P*>0.05).

**Fig. 3.**
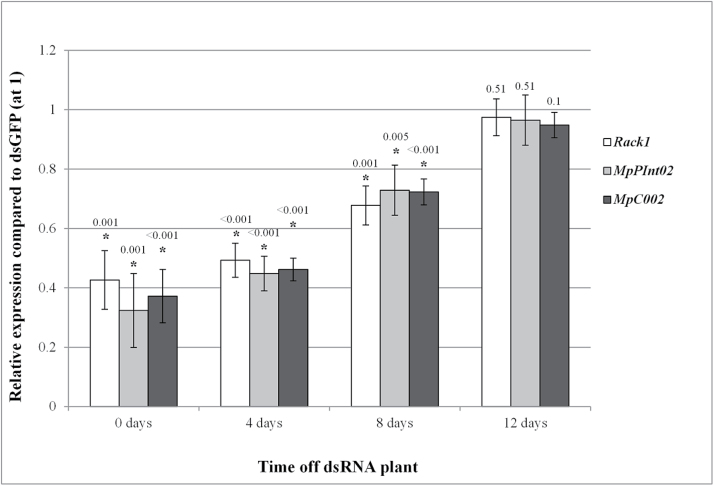
The RNAi effects spreads systemically and persist longer in aphids born from mothers exposed to the dsRNAs. RNAi GPA were transferred to Col-0 plants for 2 d to produce second-generation nymphs aged between 0 d and 2 d. Second-generation GPA were collected after 0, 4, 8, and 12 d feeding on Col-0 (0–2, 4–6, 8–10, and 12–14 d old) to test for target gene down-regulation by qRT-PCR. Relative expression of *Rack1*, *MpPIntO2*, or *MpC002* was determined in second-generation insects from RNAi aphids compared with corresponding insects produced from first-generation dsGFP-fed insects. Bars represent average expression of the corresponding target gene at each time point for progeny produced by mothers reared on dsRack1 (white), dsMpPIntO2 (grey), or dsMpC002 (black) compared with dsGFP. Data represent mean expression levels ±SD for each target gene at each time point for three biological replicates. Relative target gene expression values were normalized to the expression level of dsGFP aphids set at 1. An asterisk indicates a significant difference compared with corresponding insects from an initial dsGFP treatment (Student’s *t-*test, *n*=3, *P*<0.05), and *P*-values (to three decimal places) corresponding to individual data points are displayed above each bar.

To assess how long the RNAi effect in GPA progeny persisted, the second-generation nymphs born on wild-type plants were analysed in a longer time series. As previously, 0- to 2-day-old GPA (first generation) reared on dsRNA transgenic plants for 8 d were transferred to wild-type Col-0 plants (day 0) for 2 d, during which time they produced nymphs (second generation). The first-generation adults were removed while the second-generation nymphs remained on the plants and were harvested in three batches of five insects at 4 d intervals thereafter for qRT-PCR to assess the level of *Rack1*, *MpPIntO2*, or *MpC002* down-regulation relative to progeny produced by adult aphids fed on dsGFP transgenic plants. The second-generation aphids showed significant reductions in target gene expression levels at 0–2, 4–6, and 8–10 d old (Student’s *t-*test, *n*=3, *P*<0.0051), showing evidence of transfer of the RNAi effect from mothers to embryos (Fig. 3). Approximately 50% down-regulation was recorded for the three genes at the ages of 0–2 d and 4–6 d, and partial recoveries of gene expression levels were noticed at 8–10 d of age, while no significant level of down-regulation was observed at 12–14 d of exposure to wild-type plants (Student’s *t-*test, *n*=3, *P*>0.1) (Fig. 3). Thus, the RNAi effect lasted twice as long in second-generation GPA (up to 12 d) than in first-generation GPA (up to 6 d). Similar results were obtained for three independent biological replicates, and no statistical differences were observed among the dsRack1, dsMpPIntO2, and dsMpC002 treatments (GLM, *n*=3, *P*>0.05).

### GPA population growth is reduced on dsRNA lines

**Fig. 4.**
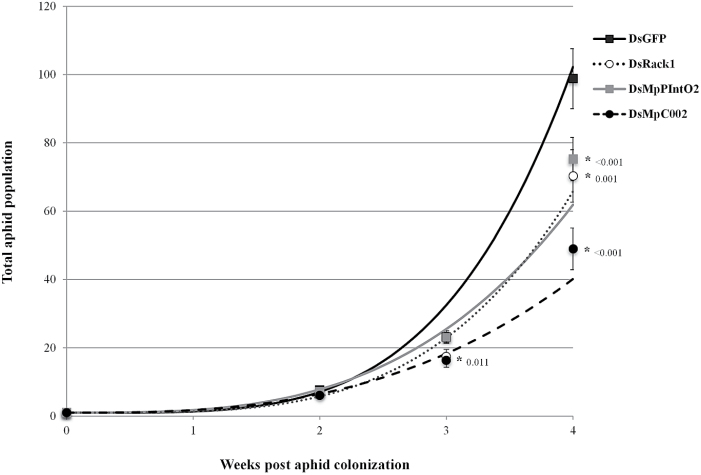
Aphid population growth is reduced by 40–60% on dsRack1, dsMpPInt02, and dsMpC002 transgenic *A. thaliana*. Aphid populations were established on dsRack1-, dsMpPInt02-, dsMpC002-, or dsGFP-expressing *A. thaliana* over 4 weeks. The mean total aphid population ±SEM from three biological replicates with *n*=4 per replicate is plotted for each time point, together with an exponential curve fitted to the data (GenStat). Curves plotted were: dsGFP, A=0.37(*t*)^4.05^; dsMpPInt02, A=1+0.76(*t*)^3.16^; dsRack1, A=1+0.35(*t*)^3.76^; dsMpC002, A=1+0.77(*t*)^2.83^ where A=total population and *t*=time in weeks. An asterisk with a corresponding *P*-value (to three decimal places) indicates a significant difference in treatments at 2, 3, or 4 weeks compared with dsGFP (Student’s *t*-test, *n*=4, *P*<0.05).

It was previously shown that plant-mediated RNAi of *Rack1*, *MpPIntO2*, and *MpC002* resulted in decreased aphid fecundity by ~10–20% at 16 d (Pitino *et al.*, 2011; Pitino and Hogenhout, 2013). Given that the RNAi effect is transferred to GPA progeny, it was hypothesized that the negative impact on fecundity of RNAi may be higher over several GPA generations. To test this, 0- to 2-day-old nymphs were seeded at one nymph per plant on four of each of the dsRack1, dsGFP, dsMpC002, and dsMpPIntO2 transgenic *A. thaliana* plants. The total numbers of adults and progeny were counted at 2, 3, and 4 weeks post-GPA inoculation (constituting about three generations of aphids). Total aphid numbers slowly declined over 4 weeks and reached a 30–40% reduction on dsMpPInt02 and dsRack1 plants and up to 60% on dsMpC002 plants compared with dsGFP plants (Fig. 4). DsRack1, dsMpC002, and dsMpPInt02 treatments all resulted in a significant reduction in the aphid population at 4 weeks (Student’s *t*-test, *n*=4, *P*<0.0009). Only dsMpC002 resulted in a significant reduction in population size by week 3 (Student’s *t*-test, *n*=4, *P*=0.011). Thus, the RNAi-mediated down-regulation of GPA genes reduces GPA fecundity over several generations and can further reduce the exponential growth of aphid populations over a longer time frame (Fig. 4). Plant-mediated RNAi of *MpC002* is more effective at reducing the GPA population than that of *Rack1* and *MpPIntO2*.

## Discussion

In this work transgenic plants producing dsRNAs corresponding to three GPA genes were used to investigate the persistence and transgenerational effects of plant-mediated RNAi in aphids. *Rack1* is a key mediator of various cellular processes inside the aphid, while candidate effector genes, *MpC002* and *MpPIntO2*, probably modulate aphid–plant interactions. Nonetheless, plant-mediated RNAi effects were similar among the three genes. Maximal reduction of gene expression was ~70%, and this was achieved at between 4 d and 8 d of exposure of the aphids to the dsRNA-producing transgenic plants. Moreover, gene expression levels returned to wild-type levels within ~6 d after removal of the aphids from the transgenic plants. Thus, gene down-regulation by plant-mediated RNAi generates consistent results and does not appear to be affected by the sequence or functions of target genes in aphids, at least for the three genes examined.

The 4–8 d period to achieve maximum gene down-regulation levels may be explained by several processes, including the time taken for the dsRNA to be taken up by the feeding aphid and for the dsRNA to be delivered to the correct target organs. At least two pathways for uptake of dsRNA in insects have been described: the transmembrane channel-mediated uptake mechanism based on *Caenorhabditis elegans* SID-1 protein (Winston *et al.*, 2007) and an ‘alternative’ endocytosis-mediated uptake mechanism (Huvenne and Smagghe, 2010; Gu and Knipple, 2013). Removal of aphids from the dsRNA-producing plants leads to recovery of gene expression to wild-type levels within 6 d. Gene knock-down also persisted for ~5 d in pea aphids fed on diets containing dsRNA (Shakesby *et al.*, 2009). Thus, continuous exposure to dsRNA is required in order to attain maximum gene knock-down in aphids. This is consistent with observations that insects lack genes encoding an RNA-dependent RNA polymerase (RdRP), the enzyme necessary for the siRNA amplification step that leads to persistent RNAi effects (Sijen *et al.*, 2001). It may therefore take ~6 d to remove all dsRNAs from aphid bodies.

It was found that target genes were also down-regulated in nymphs born from mothers exposed to dsRNA-producing transgenic plants. This was unexpected given that it is thought that insects lack genes for systemic RNAi effects (Sijen *et al.*, 2001) and embryos develop in the aphid abdomen away from the feeding mouthparts and intestinal tract, which are probably directly exposed to the dsRNAs when aphids feed from the transgenic plants. However, transfer of the silencing signal was also observed in other insect species. Parental RNAi (i.e. transfer of transcriptional down-regulation to the offspring of the target organism) has previously been reported in Coleoptera (*Tribolium castaneum*) (Bucher *et al.*, 2002), demonstrating efficient transfer of the RNAi signal across cell layers to reach germline cells. There is also recent evidence of parental transmission of RNAi in the blood-sucking Hemipteran species *Rhodnius prolixus* after injection of dsRNA (Paim *et al.*, 2013). Processes involved in systemic silencing in insects are not fully understood and there are differences between insects. For example, some insect species can be completely refractory to systemic RNAi, whereas close to 100% knock-down can be achieved in others (Belles, 2010). The precise mechanism of how systemic RNAi is achieved in aphids remains to be elucidated. Nonetheless, it is clear that dsRNAs are required to maintain the RNAi effects, indicating that, while these effects are systemic, they are not persistent in aphids.

Another unexpected finding was that the RNAi effect lasted twice as long (12 d) in nymphs born from mothers exposed to dsRNA-producing transgenic plants. Thus, the RNAi effect is more persistent in the nymphs than in their mothers. Multiple factors may affect dsRNA stability in insects, including dsRNA concentrations, lengths of dsRNA fragments, dsRNA degradation activities, and the life stages of the target organisms (Griebler *et al.*, 2008; Huvenne and Smagghe, 2010; Bolognesi *et al.*, 2012; Kumar *et al.*, 2012; Miller *et al.*, 2012). Furthermore, there appears to be stronger dsRNase activity in certain tissue types. In the pea aphid, both the salivary secretions and the haemolymph of aphids are able to degrade dsRNA (Christiaens *et al.*, 2014), and potent dsRNA-degrading activity was found in the midgut juice of the desert locust (*Schistocerca gregaria*), resulting in lower sensitivity to RNAi via oral administration of dsRNA compared with microinjection (Wynant *et al.*, 2014). In the present experiments, first-generation GPA acquired dsRNAs via the oral route by feeding from the dsRNA-producing plants, while the second-generation nymphs were exposed to the dsRNAs via their mothers. The different dsRNA acquisition routes between mothers and daughters may alter concentrations and distributions of dsRNAs in organ and tissue types that affect dsRNA stability, leading to differences in RNAi persistence between the two generations. Nevertheless, the finding that the RNAi effect is persistent and transferred to the next generation in aphids renders plant-mediated RNAi as a powerful tool to control aphid outbreaks in the field.

In the present RNAi experiments, the maximal reduction of gene expression was ~70%. Thus, complete knock-down of gene expression levels was not achieved. It is possible that the dsRNAs do not reach all tissues in the aphid and that these unreachable tissues accumulate to a basal gene expression level of 30%. However, it was previously observed that *Rack1*, *MpC002*, and *MpPIntO2* are expressed in different aphid organs (Pitino *et al.*, 2011). If the dsRNAs only reach a few body parts, one would expect to see differences in the levels of down-regulation between these genes in the RNAi experiments. Nonetheless, complete knock-down of *C002* in *A. pisum* was achieved by injecting dsRNA corresponding to C002 into the haemolymph after 4 d (Mutti *et al.*, 2006). Together, the data suggest that dose and site of exposure probably affect RNAi efficiencies.

Reducing the expression of *Rack1*, *MpC002*, and *MpPIntO2* has a profound negative effect on aphid reproduction levels. An ~10–20% decrease in aphid reproduction was previously found within one generation of aphids (Pitino *et al.*, 2011; Pitino and Hogenhout, 2013). Here it is shown that the negative effects on aphid reproduction become clearer when observing multiple aphid generations. Aphid populations grow exponentially, as observed in the dsGFP control treatment, but RNAi of *MpC002* causes a 60% decline in the aphid population growth compared with 40% of *Rack1* and *MpPIntO2*. This confirms previous assessments that C002 has an important function in aphids (Mutti *et al.*, 2008; Bos *et al.*, 2010), more so than a conserved multifunctional gene, such as *Rack1*, which has important functions in a number of organisms; knock-down of *Rack1* results in developmentally defective phenotypes in *C. elegans* (Kamath *et al.*, 2003; Simmer *et al.*, 2003; Ciche and Sternberg, 2007) and *Drosophila melanogaster* (Kadrmas *et al.*, 2007; Kucherenko *et al.*, 2008). The impact of down-regulation of *MpPIntO2* is similar to that of *Rack1*, indicating that MpPIntO2 also has important functions in GPA.

Aphids are among the most important insect pests of arable crops in temperate regions worldwide (Blackman and Eastop, 2000). As well as causing direct feeding damage to crops, aphids are capable of transmitting ~30% of the plant virus species discovered to date (Ng and Perry, 2004; Hogenhout *et al.*, 2008). A reduction of the aphid reproduction by 40–60% achieved by plant-mediated RNAi of *MpC002*, *MpPIntO2*, and *Rack1* would dramatically decrease aphid population growth, leading to a substantial reduction in agricultural losses.
